# Effects of 8 months of high-intensity interval training on physical fitness and health-related quality of life in substance use disorder

**DOI:** 10.3389/fpsyt.2023.1093106

**Published:** 2023-08-09

**Authors:** Jun Tan, Jingsong Wang, Yin Guo, Chunxia Lu, Wanke Tang, Lan Zheng

**Affiliations:** ^1^Hunan Normal University, Changsha, Hunan, China; ^2^Hunan International Economics University, Changsha, Hunan, China; ^3^Fujian Normal University, Fuzhou, Fujian, China

**Keywords:** high-intensity interval training, physical fitness, health-related quality of life, abstinent drug subjects, exercise

## Abstract

**Objective:**

This study aimed to investigate the effect of 8 months of high-intensity interval training (HIIT) on physical fitness and health-related quality of life in substance use disorder.

**Methods:**

Sixty substance use disorder were randomly assigned to either the HIIT group or the control group according to a random sampling method. The HIIT group received 8 months of four 60-min sessions per week under supervision. Weight, waist circumference, body fat percentage, heart rate, blood pressure, VO2max, reaction time, grip strength, standing on one foot with eyes closed, sitting forward flexion, and quadrant jumping, standing on one foot with eyes closed, the number of push-ups, quality of life (SF-36) score, and craving (VAS) scored were monitored in the HIIT and control groups at baseline, 4 months, and 8 months. SPSS 22.0 was used to conduct repeated measurement analysis of variance and Pearson correlation analysis on the collected subject data.

**Results:**

Compared with baseline, weight (*p* < 0.001), waist circumference (*p* < 0.001), body fat percentage (*p* < 0.001), heart rate (*p* < 0.05), Systolic blood pressure (*p* < 0.01), systolic blood pressure (*p* < 0.05), reaction time (*p* < 0.001),PSQI (*p < 0.*001), Total cholesterol (*p < 0.*001), Triglyceride (*p < 0.*001), Blood sugar (*p < 0.*001) and VAS score (*p* < 0.001) were significantly decreased after 8 months of exercise intervention. Contrastingly, VO2max (*p* < 0.05), grip strength (*p* < 0.05), eyes closed and one foot Standing (*p* < 0.001), sitting forward flexion (*p* < 0.001), quadrant jumping (*p* < 0.001), push-ups (*p* < 0.001), PCS (*p* < 0.001), and MCS (*p* < 0.001) were significantly increased. VO2max was significantly negatively correlated with VAS (*r* = −0.434, *p* < 0.001), and significantly positively correlated with PCS (*r* = 0.425, *p* < 0.001). There was a positive correlation between standing on one foot with closed eyes and MCS (*r* = 0.283, *p* < 0.05).

**Conclusion:**

Eight months of HIIT can comprehensively improve the physical health level and health-related quality of life of men with substance use disorders, reduce the desire for drugs, and lay the foundation for better starting a happy life.

## Introduction

Drugs pose a considerable threat to the safety and health of the world’s citizens. By the end of 2021, 1.486 million existing person who uses drugs have been registered in China, 121,000 more person who uses drugs than before (1). Indeed, China’s social security is gravely threatened by drug abuse, the number one public enemy. Its high rate has resulted in the Chinese government investing enormously in manpower and material resources.

Substance abuse is considered a chronic, relapsing disorder (2), and the most prevalent issues faced by substance abusers are a lower quality of life and a shortened life expectancy, which are not only lower than normal but comparable to those with other severe mental illnesses (3–6). At the same time, low quality of life is also closely related to a higher relapse rate in drug abusers (7). The traditional view is that the withdrawal from addiction is chiefly characterized by drug withdrawal, yet it is well documented that active treatment of individuals, as well as recovery outcomes, are critical to the final evaluation of addiction (5, 8–11).

Substance abusers frequently suffer from psychiatric, physical, and social comorbidities, in addition to chronic, relapsing diseases, leading to a reduced life expectancy (12). Meanwhile, substance abusers have a low health-related quality of life (HRQoL) and high chronic disease severity and psychiatric comorbidities as in patients with similar diseases (13–15). Assessment of health-related quality of life (HRQoL) is gradually being recognized as a key parameter for assessing substance abuse treatment (5, 10, 11). Modulating the detrimental effects of substance abuse (16, 17), as well as enhancing self-efficacy and other psychological structures (18), have been proposed as mechanisms for the treatment of drug addiction.

Previous studies have established that long-term drug use severely impairs aerobic endurance and muscle strength in patients (19). As expected, drug withdrawal patients have less muscle protein and mass than healthy individuals and are prone to hypertensive reflexes, dyskinesia, and gait instability (20). Additionally, conditions such as decreased physical fitness, tachycardia, hypertension (BP), and chronic cardiovascular disease may also arise (21). The combined effect of drugs on physical and mental health not only reduces the quality of life but also contributes to the difficulty of withdrawal. Therefore, there is an urgent need to identify an interventional strategy to promote the physical and mental rehabilitation of patients experiencing drug withdrawal.

Exercise is progressively being integrated into the field of drug addiction as a green and healthy behavioral intervention (22, 23). It not only reduces peripheral inflammation in substance use disorder but also modulates plasma oxytocin levels and improves social anxiety and cue-induced craving in heroin addicts (22, 23). Although exercise is extensively used as an intervention in drug withdrawal patients and exerts long-lasting therapeutic effects, the majority of studies on exercise detoxification have focused on peripheral blood markers, and studies focusing on physical and mental rehabilitation effects are limited, especially studies on the mechanism of exercise and improvements in quality of life from the standpoint of physical fitness in drug withdrawal patients. Some scholars have even recommended research methodologies on the effects of modulating chronic physical health conditions and improving self-efficacy and mental structure on the quality of life (16–18).

In recent years, HIIT has emerged as an alternative or complementary form of training to aerobic exercise. In both long-term and short-term studies, HIIT is as valuable or even superior to aerobic exercise in terms of short-term fitness, cardiovascular function, quality of life, exercise efficiency, safety, tolerance, and exercise compliance (24). Earlier studies have shown that compared with aerobic exercise, HIIT can significantly improve the physical fitness and craving of substance use disorder (25). At the same time, HIIT is also widely considered to be the preferred intervention to improve the quality of life of several chronic disease populations (26–28). Therefore, the main purpose of this study was to investigate the effect of HIIT on improvements in physical fitness level and health-related quality of life of drug withdrawal patients and to observe the sleep quality, cue-induced craving, blood sugar and lipid levels, etc., of drug withdrawal patients as auxiliary indicators. We hypothesized that HIIT can improve physical fitness levels in drug abstainers while improving health-related quality of life and reducing cue-induced cravings. The findings of this study may provide a guide and reference for substance use disorder to have a healthy lifestyle and a fresh start.

## Study subjects and methods

### Subjects

One thousand two hundred and sixty male compulsory isolation and detoxification personnel from the Bainihu Compulsory Isolation and Rehabilitation Center in Hunan Province were enrolled in this study. The screening criteria were as follows:

(1) All participants met the criteria for MA dependence in accordance with the structured diagnostic criteria of DSM-V; (2) patients not on other drugs; (3) The way of taking drug is scalding; (4) no history of mental illnesses; (5) No history of skeletal, muscular diseases, cardiovascular, cerebrovascular, or immune diseases; (6) No history of major trauma and surgery; (7) Alcohol, nicotine, caffeine, and β-blockers were not consumed within 6 h of the cardiopulmonary function test (8). No regular exercise habits (defined as self-reported moderate-intensity physical activity within three months not exceeding 30 min per day and less than three days per week); (9) Patients who consented to complete the PAR-Q+ (Physical Activity Readiness Questionnaire).

The study plan was approved by the Ethical Review Committee of Hunan Normal University (batch number: 2020–233) and was conducted in accordance with the Declaration of Helsinki. All 60 included subjects signed the informed consent and voluntarily participated in this trial. At the time of issuing the informed consent form, we have stated to the subject that we can terminate the experiment at any time without any punishment or loss. The 60 substance use disorder were divided into the exercise group and the control group according to the random sampling method. There was no significant difference in the duration, frequency, and amount of drug abuse between the two groups (*p* > 0.05). As presented in Table 1, two patients in the exercise group (withdrawal rate 6.7%) withdrew from the study following injury, whereas 2 participants in the control group (withdrawal rate 6.7%) withdrew for personal family reasons:

**Figure 1:**
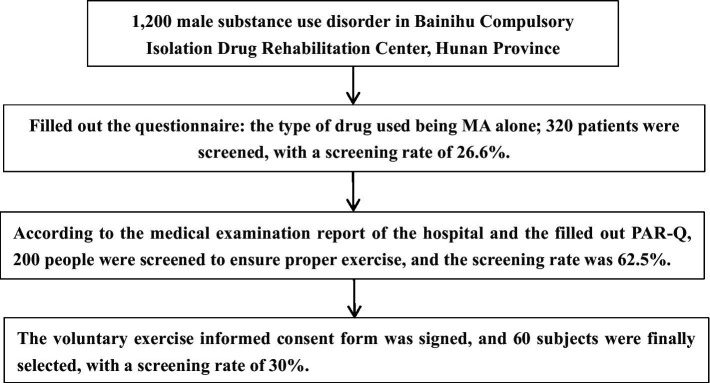
Screening flow chart.

**Table 1:** Baseline characteristics.

Content	Exercise group (*n* = 30)	Control group (*n* = 30)
Age (year)	32.25 ± 6.29	31.86 ± 4.99
Height (cm)	166.46 ± 5.06	168.04 ± 7.06
Weight (kg)	66.04 ± 8.01	67.07 ± 9.21
Drug history (month)	69.43 ± 30.98	53.25 ± 36.07
Frequency of drug (week)	11.50 ± 6.84	9.50 ± 8.44
Intensity per time (g)	0.54 ± 0.42	0.42 ± 0.38

### Outcome measurements and experimental procedures

All primary and secondary endpoints were measured before and after the exercise intervention. The primary objectives included body circumference and composition, heart rate, blood pressure, muscle strength, explosive power, cardiopulmonary aerobic fitness, balance, sensitivity, flexibility, speed, coordination, etc. HRQol was determined by SF-36, and the secondary endpoints were the degree of drug craving measured by VAS. Prior to the test, participants did not consume food and beverages other than water for 12 h, and all tests except the pretest were conducted on the second day following the previous training session.

Main indicators:

### Resting heart rate and blood pressure

Resting heart rate and blood pressure were scheduled to be the first measurements after waking up in the morning (6.00 a.m.). Subjects sat quietly on a chair for 5 min with their back supported on a chair, feet flat on the floor, and arms supported at heart level. The doctor utilized a sphygmomanometer (OMRON T10) to measure the subjects’ resting heart rate and systolic and diastolic blood pressure.

### Anthropometric measurements

Subjects measured their body weight on a medical scale accurate to 0.1 kg. Body fat percentage was determined by a body fat measuring instrument (Belida BC-401) with an accuracy of ±100 g. The waist circumference was measured with a soft ruler at the horizontal girth through the center of the umbilicus, or the girth of the midpoint line between the lowest point of the rib and the upper border of the iliac crest, at the end of exhalation and before inhalation.

### VO_2_ Max

Participants completed an incremental load exercise test of the modified Bruce protocol (Table 2) on a treadmill (Cosmos Pulsar 4.0) and adjusted the speed and inclination level (increasing after level 7) according to the protocol until volitional fatigue. Subjective perception of effort (RPE) was measured at the last minute of each load level. The termination criteria of the incremental stress exercise test were: aberrant electrocardiogram, reaching personal maximum heart rate, abnormal blood pressure, RPE greater than 17, respiratory quotient greater than 1.15, etc. Subjects were asked about their subjective feeling of fatigue at any time during the test. Participants’ HR was continuously recorded during the test by 12-lead ECG, chest strap (Polar V800), cortex gas metabolism analyzer, and radio telemetry-receiving equipment. The subjects wore breathing masks, cooperated with cardiopulmonary function testing (MetaLyzer II-R2) and training equipment (HP Cosmos Pulsar) to complete the VO2max test, and the actual maximum heart rate (Bpm) was recorded.

**Table 2:** Quality of life, cue-induced craving outcomes at baseline, 4 months, and 8 months in the Control and HIIT groups.

	Control group (*n* = 28) baseline4 months8 months	HIIT group (*n* = 28) baseline4 months8 months	*p* (intergroup)	*p* (time)	*p* (interaction)
PF (score)	75.60 ± 22.90 75.63 ± 20.31 75.12 ± 20.08	73.21 ± 20.14 74.21 ± 21.89 76.25 ± 22.45****△△	<0.05	<0.001	<0.001
RP (score)	62.80 ± 23.45 62.58 ± 23.18 62.00 ± 23.78	60.34 ± 21.47 61.35 ± 20.14 63.47 ± 21.61**	0.421	<0.01	<0.001
BP (score)	70.40 ± 22.32 70.12 ± 23.65 68.32 ± 25.41	66.45 ± 20.31 66.19 ± 22.34 66.97 ± 21.45	0.547	<0.05	<0.01
GH (score)	51.44 ± 15.91 51.41 ± 15.74 50.19 ± 15.71	49.14 ± 13.95 51.45 ± 15.87 54.83 ± 15.47****△△	<0.01	<0.001	<0.001
PCS (score)	65.06 ± 11.87 65.11 ± 12.20 64.83 ± 12.49	62.25 ± 14.85 63.48 ± 14.66 66.12 ± 12.73****△△	0.764	<0.001	<0.001
VI (score)	50.51 ± 14.82 50.42 ± 14.29 50.74 ± 14.08	53.43 ± 15.47 53.45 ± 16.93 54.42 ± 15.74	0.054	<0.05	<0.001
SF (score)	60.35 ± 20.47 60.34 ± 20.17 60.72 ± 20.67	62.72 ± 21.49 63.24 ± 20.78 63.47 ± 16.37* **△△▲	<0.01	<0.001	<0.001
RE (score)	59.07 ± 23.86 59.01 ± 23.47 59.38 ± 23.47	62.25 ± 24.27 62.47 ± 20.78 63.78 ± 22.47	0.074	<0.05	<0.01
MH (score)	47.95 ± 14.42 47.85 ± 14.03 47.23 ± 14.81	53.81 ± 16.74 53.71 ± 15.47 54.74 ± 18.13**△△▲▲	<0.01	<0.001	<0.001
MCS (score)	54.47 ± 8.97 54.67 ± 8.62 54.69 ± 8.84	58.05 ± 10.40 59.06 ± 10.67 60.10 ± 9.98* **△△▲	0.083	<0.001	<0.01
VAS (score)	32.00 ± 24.08 25.71 ± 24.26 22.39 ± 19.08	33.21 ± 28.03 16.82 ± 17.49 11.25 ± 13.31**▲	0.079	<0.001	0.258

*p < 0.05, compared with baseline; **p < 0.01, compared with baseline; △*p* < 0.05, compared with 4 months; △△*p* < 0.01, compared with 4 months; ▲*p* < 0.05, compared with the control group.

### Grip strength, push-up test

Grip strength and push-ups were employed for muscle strength testing. During the hand-grip test, subjects were instructed to hold the gripping device for a few seconds with the maximum force expressed in kilograms (kg). The 1-min push-up test was performed on a push-up tester with the subject’s front feet or toes on the floor, the hips and back straight, and the push-up tester placed near the subject’s chest. Whenever the torso moved up and down in front of the tester’s sensor, the push-up tester automatically counted the number of repetitions.

### Reaction time

A reaction time instrument was employed to measure reaction speed. The subject was instructed to press the button with his/her right thumb when the light went on, and the mean of three measurements was recorded in milliseconds (ms).

### Stand on one foot with eyes closed

The one-legged standing balance test is a simple method for measuring static balance. The individual balances on one leg with the eyes closed and relies on the balance receptors of the vestibular organs of the brain and the joint coordination of the muscles of the whole body to maintain body balance. The length of balancing is directly proportional to the static balance ability.

### Sitting forward bend

The patients were instructed to extend their legs with their heels touching and toes naturally separated. Then, with the palms facing downward, the arm extended, and the upper body bent forward, the patients were directed to move two fingers forward at a constant speed until they were unable to continue. The test was conducted three times, and the maximum distance reachable with the subject’s fingertips was measured in centimeters (cm), and the average was calculated.

### Quadrant jump

A sensitivity quality test was used to measure 10 s quadrant jumps. The patients were instructed to maintain a natural stance with their feet together, back straight, and hands on hips. Next, the patients were commanded to jump in four directions, and the number of completed quadrants within 10 s was counted.

### 36-item brief health survey (SF-36 v2.0)

The SF-36 tool was used to analyze health-related quality of life (HRQoL). It consists of 36 items comprising 8 dimensions and 1 health change item. The eight dimensions are physical functioning (PF), role-physical (RP), bodily-pain (BP), general health (GH), vitality (VT), social function (Social Functioning, SF), emotional function (Role-Emotional. RE), and mental health (Mental Health, MH). It also includes a health change entry that is not factored into the calculation. The survey combines individual parameters into groups: four parameters for the physical domain assessment and four parameters for the psychological domain for quality of life. The following assignments were made: PF + RF + BT + GH + Subjective perception of health change = Body Composition Summary (PCS); VT + SF + RE + MH = Mental Composition Summary (MCS). Scores for each dimension were calculated according to the handbook, with scores ranging from 0 to 100 for each dimension; scores closer to 100 indicated a higher quality of life.

Secondary indicator.

### Visual analog scale

VAS is used to visually assess the immediate desire for MA by patients. A 0–100 mm VAS scale was adopted for determining the degree of cue-induced craving (0 indicates “no craving”; 100 indicates “extreme craving”). The patient was requested to relax for 5 min and then watch neutral pictures and videos for 5 min. Following this, the patient was requested to watch pictures and videos of objects, utensils, and MA inhalation for 5 min. The VAS score was calculated immediately after the induction process. Psychological scale assessments and blood index testing were conducted a day before conducting the exercise intervention and a day after termination of the exercise intervention.

### Pittsburgh sleep quality index scale

Subjects filled out this scale and assessed their sleep quality through subjective scores. The Pittsburgh Sleep Quality Index Scale (29) was used in this study, which is used to assess the subjective sleep quality of the subjects during the past month. A degree test is considered suitable for domestic patients. The PSQI scale is divided into seven factors, each of which is divided into four grades, with the lowest score being 0 and the highest being 3. The higher the total score, the worse the sleep quality.

### Measurement of fasting blood lipids and glucose

Subjects took venous blood without breakfast in the morning (7:00–8:00 a.m.). All fasting blood lipids and blood glucose were analyzed in duplicate at room temperature. Take two tubes of 3 mL venous blood and take the average value as the final result. The content of determination includes: total cholesterol, triglyceride, and blood sugar.

### Exercise intervention

The exercise group underwent high-intensity intermittent exercise to intervene in methamphetamine withdrawal, and the control group received routine rehabilitation therapy. Exercise group training content included non-confrontational basketball training, resistance exercises (strength equipment training), running, and skipping rope. The exercise course lasted 60 min each session, 4 times a week, for a total of 8 months, including 10 min of warm-up exercise, 40 min of basic content exercises, and 10 min of stretching and relaxation exercises. Group heart rate monitoring equipment (Polar Team Pro) was used to monitor heart rate intensity. Supervisors urged trainers to maintain the HR at 76–96% HRmax of the subjects (the maximum heart rate of each person was determined with the VO2max test before the exercise session), allowing HR responses to fluctuate within the target HR range. The average heart rate was maintained at 150–170 beats per minute during training and 120–140 during recovery. The train-to-recovery ratio of subjects during training was 1:0.5. The training mode was organized, supervised, and taught by doctoral and master students majoring in sports human science and sports training at the School of Physical Education of Hunan Normal University.

### Statistical methods

Baseline, 4-month, and 8-month data were analyzed using repeated measures ANOVA. ANOVA was used to compare multiple groups, and if *p* < 0.05, the Bonferroni method was used for pairwise comparison between groups. Statistical analysis was performed using SPSS 20.0 software, and experimental data were expressed as mean ± standard deviation (M ± SD); graphical presentations were made using GraphPad Prism 5 (GraphPad Software, San Diego, CA, USA).

## Results

Physical and physiological outcomes of substance use disorder at baseline, 4 months, and 8 months. At baseline, there was no difference between the control group and the HIIT group in weight, waist circumference, body fat percentage, heart rate, blood pressure, VO2max, reaction time, grip strength, standing on one foot with eyes closed, sitting forward flexion, quadrant jumping, PSQI, Total cholesterol, Triglyceride, and Blood sugar. There were differences between push-ups and eyes-closed standing on one foot. In the 8-month time × group interaction, differences in weight (*F* = 13.103, df = 2, partial η^2^ = 0.325), waist circumference (*F* = 15.977, df = 2, partial η^2^ = 0.000), body fat percentage (*F* = 75.925, df = 2, partial η^2^ = 0.671), heart rate (*F* = 13.103, df = 2, partial η^2^ = 0.229), diastolic blood pressure (*F* = 0.082, df = 2, partial η^2^ = 0.291), VO2max (*F* = 7.767, df = 2, partial η^2^ = 0.227), reaction time (*F* = 27.093, df = 2, partial η^2^ = 0.334), grip strength (*F* = 5.072, df = 2, partial η^2^ = 0.161), standing on one foot with eyes closed (*F* = 10.479, df = 2, partial η^2^ = 0.307), sitting forward bend (*F* = 33.597, df = 2, partial η^2^ = 0.660), quadrant jump (*F* = 20.344, df = 2, partial η^2^ = 0.613), PSQI (*F* = 9.21，df = 2, partial η^2^ = 0.207)and push-up (*F* = 16.475, df = 2, partial η^2^ = 0.383),Total cholesterol (*F* = 5.87, df = 2, partial η^2^ = 0.460),Triglyceride (*F* = 3.65, df = 2, partial η^2^ = 0.145) and Blood sugar (*F* = 36.18, df = 2, partial η^2^ = 0.324)were significant, whereas the difference in systolic blood pressure was not (*F* = 2.802, df = 2, partial η^2^ = 0.068). After four months of exercise intervention, the waist circumference (*p* < 0.05, 95% CI: 0.071, 2.643), BMI (*p* < 0.001, 95% CI: 0.709, 1.698), heart rate (*p* < 0.001, 95% CI: 1.135, 3.793), systolic blood pressure (*p* < 0.05, 95% CI: 1.14, 2.601), diastolic blood pressure (*p* < 0.01, 95% CI: 0.614, 3.171), triglycerides (*p* < 0.01, 95% CI: −4.184, −2.601), blood glucose (p < 0.05, 95% CI: 1.198, 2.701), reaction time (*p* < 0.001, 95% CI: 7.510, 23.490), and PSQI (*p* < 0.05, 95% CI: 1.98, 3.874) of HIIT group decreased, but VO2max (*p* < 0.001, 95% CI: −1.949, −0.394), push up (*p* < 0.001, 95% CI: −8.959, −2.827), sitting forward bending (*p* < 0.001, 95% CI: −4.241, −2.181), quadrant jump (*p* < 0.001, 95% CI: −4.476, −3.024) increased. After 8 months of exercise intervention, weight (*p* < 0.001, 95% CI: 0.427, 1.479), waist circumference (*p* < 0.001, 95% CI: 1.407, 4.236), body fat percentage (*p* < 0.001, 95% CI: 1.350, 2.608), heart rate (*p* < 0.05, 95% CI: 0.167, 1.690), reaction time (*p* < 0.001, 95% CI: 56.208, 123.507) and diastolic (*p* < 0.01, 95% CI: 0.594, 4.334) and systolic blood pressure (*p* < 0.01, 95% CI: 0.594, 4.334; 0.05, 95% CI: 0.234, 3.194) were significantly decreased in the HIIT group compared with baseline levels, whereas VO2max (*p* < 0.05, 95% CI: −4.902, −0.119), grip strength (*p* < 0.001, 95% CI: 56.208, 123.507), standing on one foot with eyes closed (*p* < 0.001, 95% CI: −25.352, −10.934), sitting forward flexion (*p* < 0.001, 95% CI: −4.502, −2.162), quadrant jumps (*p* < 0.001, 95% CI: −6.737, −4.049), and push-ups (*p* < 0.001, 95% CI: −17.6846, −7.530) were significantly increased. Compared with the control group, substance use disorder in the HIIT group exhibited significantly higher waist circumference (*p* < 0.01, 95% CI: −9.582, −2.661), while body fat percentage (*p* < 0.001, 95% CI: −4.938, −2.219), PSQI (*p < 0.*001, 95% CI: 1.21, 3.26), Total cholesterol (*p < 0.*001, 95% CI:–7.25, −2.47), Triglyceride (*p < 0.*001, 95% CI: −5.18, −2.90), Blood sugar (*p < 0.*001, 95% CI: 1.18, 3.45) was significantly decreased at 8 months. Meanwhile, VO2max (*p* < 0.05, 95% CI: 1.041, 7.934), push-ups (p < 0.001, 95% CI: −6.737, −4.049), reaction time (*p* < 0.001, 95% CI: −105.164, −39.121), standing with eyes closed (*p* < 0.01, 95% CI: 9.137, 31.291), sitting forward flexion (*p* < 0.001, 95% CI: 7.347, 12.581), and quadrant jumping (*p* < 0.01, 95% CI: 1.083, 4.560) were significantly higher than substance use disorder in the control group (Table 3).

**Table 3:** Physical and physiological outcomes of patients in the control and HIIT groups at baseline, 4 months, and 8 month.

	Control group (*n* = 28) baseline4 months8 months	HIIT group (*n* = 28) baseline4 months8 months	*p* (intergroup)	*p* (time)	*p* (interaction)
Weight (kg)	67.73 ± 7.68 67.66 ± 8.35 68.28 ± 7.93	67.34 ± 7.99 67.23 ± 8.24 66.39 ± 8.60**△	0.677	0.414	<0.001
Waistline (cm)	83.32 ± 9.51 84.46 ± 9.34 84.39 ± 8.47	81.39 ± 4.12 80.04 ± 4.73 78.57 ± 5.17*▲**△△▲▲	<0.05	<0.05	<0.001
BMI (%)	20.49 ± 2.74 21.70 ± 2.41 18.65 ± 2.92 ** **△	20.63 ± 3.17 19.43 ± 3.14 18.65 ± 2.92**▲▲**△△▲▲	<0.05	0.657	<0.001
Heart rate (bmp)	73.4 ± 9.88 73.64 ± 9.74 73.04 ± 9.61	73.75 ± 7.16 71.29 ± 7.43 70.36 ± 7.72****△	0.52	<0.001	<0.01
Grip (kg)	42.43 ± 8.47 41.73 ± 7.34 41.20 ± 6.57	39.66 ± 6.28 40.33 ± 6.20 41.82 ± 6.64*△△	0.514	0.156	<0.01
Systolic blood pressure (mmHg)	117.18 ± 5.64116.79 ± 5.44117.11 ± 5.88	119.21 ± 5.16117.86 ± 6.16117.50 ± 5.47**	0.425	<0.05	0.082
Diastolic blood pressure (mmHg)	73.82 ± 7.29 75.18 ± 8.75 74.82 ± 8.91	75.50 ± 7.23 73.61 ± 6.76 73.04 ± 6.24 ** **	0.776	0.301	<0.01
VO_2_max (ml/kg/min)	35.90 ± 5.32 35.51 ± 5.41 34.51 ± 8.03	36.49 ± 4.97 37.66 ± 4.29 39.00 ± 4.26** *▲	0.075	0.227	<0.01
Push ups (number)	34.25 ± 7.69 33.61 ± 7.96 31.61 ± 10.43	46.75 ± 11.29 52.64 ± 10.42 59.36 ± 11.94▲▲ **▲▲ **△△▲▲	<0.001	<0.01	<0.001
Reaction time (ms)	506.96 ± 6.52516.57 ± 60.46530.00 ± 71.77	547.71 ± 99.33532.21 ± 102.11457.86 ± 49.44**▲▲ **△△▲▲	0.773	<0.001	<0.001
Standing with eyes closed(s)	11.14 ± 16.06 14.25 ± 18.45 13.39 ± 15.73*	15.46 ± 11.76 23.82 ± 17.43 33.61 ± 24.64** **△△▲▲	0.10	<0.001	<0.001
Sitting forward bend (cm)	7.48 ± 4.18 6.64 ± 4.22 5.18 ± 3.70**	11.81 ± 4.69 15.03 ± 5.15 15.15 ± 5.84▲▲ **▲▲ **▲▲	<0.001	<0.01	<0.001
Quadrant jump (number)	23.86 ± 3.92 24.18 ± 3.99 25.11 ± 3.06	22.54 ± 2.74 26.29 ± 3.33 27.93 ± 3.42**▲ **△△▲▲	0.152	<0.001	<0.001
Total cholesterol (mmol/L)	4.93 ± 0.68 4.53 ± 0.65 4.63 ± 0.56*	4.71 ± 0.66 4.31 ± 0.47 3.64 ± 0.59**△△▲▲	<0.001	<0.01	<0.001
Triglyceride (mg/dL)	7.03 ± 0.65 6.87 ± 0.73 6.62 ± 0.87	74.81 ± 6.23 68.57 ± 7.40 62.55 ± 6.66**▲ **△▲▲	<0.001	<0.001	<0.001
Blood sugar (mmol/L)	7.03 ± 0.65 6.87 ± 0.73 6.62 ± 0.87	7.37 ± 0.65 6.40 ± 0.80 5.74 ± 0.62** **△△▲▲	<0.001	<0.01	<0.001
PSQI (score)	12.50 ± 3.87 12.48 ± 3.91 12.62 ± 3.22	12.25 ± 4.03 10.96 ± 3.87 9.98 ± 3.26**▲▲ **△△▲▲	<0.001	0.136	<0.001

*p < 0.05, compared with baseline; **p < 0.01, compared with baseline; △*p* < 0.05, compared with 4 months; △△*p* < 0.01, compared with 4 months; ▲*p* < 0.05, compared with the control group; ▲▲*p* < 0.01, compared with the control group.

At baseline, there were no significant between-group differences in PCS, MCS, and VAS scores between the control group and the HIIT group. In the interaction effect of time × group at 8 months, the interaction effect of PCS (*F* = 11.157, df = 2, partial η^2^ = 0.212) and MCS (*F* = 7.542, df = 2, partial η^2^ = 0.200) was significant, whereas that of VAS (*F* = 1.373, df = 2, partial η^2^ = 0.046) was not. After 4 and 8 months of exercise intervention, PCS (*p* < 0.001, 95% CI: 0.427, 1.479) and MCS (*p* < 0.001, 95% CI: 1.407, 4.236) were significantly increased in the HIIT group decreased significantly，VAS (*p* < 0.001, 95% CI: 8.167, 35.762) increased significantly. Compared with the baseline level, after 8 months of exercise intervention, the MCS (*p* < 0.05, 95% CI: 0.358, 10.462) of the HIIT group was significantly higher than that of the control group, while VAS (*p* < 0.05, 95% CI: −19.956, −2.329) was significantly lower (Table 3).

### Correlation analysis of cue-induced craving and health-related quality of life of various physiological indicators

Through Pearson correlation analysis of various physiological indicators with craving degree and quality of life, it was found that VO2max was significantly negatively correlated with VAS (*r* = −0.434, *p* < 0.001), and significantly positively correlated with PCS (*r* = 0.425, *p* < 0.001). There was a significant positive correlation between monocular standing with closed eyes and MCS (*r* = 0.283, *p* < 0.05). There was a significant positive correlation between MCS and PCS (*r* = 0.459, *p* < 0.001; Supplementary Table 1).

## Discussion

To the best of our knowledge, this is the first study to investigate the effect of HIIT on physical fitness combined with health-related quality of life parameters in substance use disorder, and the physical fitness indicators involved were relatively comprehensive, including not only speed, strength, endurance, agility, flexibility, and balance but also included various physiological indicators (heart rate and blood pressure) and health-related morphological indicators (weight, body fat rate, and waist circumference). Our study supports the hypothesis that 8 months of HIIT not only improves health-related quality of life in methamphetamine addicts but also significantly improves speed, strength, endurance, agility, flexibility, balance, weight control, and lowers the risk of cardiovascular diseases.

### Improvement in the cardiovascular system and cardiorespiratory fitness

Substance abuse impairs cardiac autonomic regulation, and drug abuse has been shown to increase adrenergic activity and decrease vagal tone in the cardiovascular system, leading to worse heart rate variability and increased risk of death (30–32). In our study, 8-month HIIT significantly reduced heart rate, systolic blood pressure and diastolic blood pressure, and in terms of biochemical indicators, 8-month HIIT significantly reduced subjects’ triglycerides, total cholesterol and blood sugar, which to some extent showed that exercise improved the cardiovascular system function of patients with substance use disorders and reduced the risk of cardiovascular disease. A cross-sectional study of stimulant-using patients determined that their cardiorespiratory fitness levels were generally lower (33). Prior studies of methamphetamine users have also reported cardiorespiratory fitness levels well below the population average (19, 34). However, some studies have validated that exercise can improve cardiorespiratory fitness and increase vagal autonomic activity to limit injuries caused by drug abuse (35). The present study revealed that 8 months of HIIT exercise increased VO2max and decreased resting heart rate and systolic and diastolic blood pressure in abstinent methamphetamine subjects, in line with findings of previous studies. A possible mechanism underlying this observation is that HIIT includes high-intensity aerobic running, which can increase the VO2max while strengthening the tone of the parasympathetic nervous system, reducing the tone of the sympathetic nervous system and increasing the autonomic activity of the vagus nerve, resulting in a lower resting heart rate.

### Improvement in response time

Neuroimaging studies have established that drug addiction severely disrupts response inhibition in humans, resulting in decreased response flexibility (36). However, most studies have focused on decreased thalamic activation in response to drug cues (37). Earlier studies have also uncovered that high-intensity interval and moderate-intensity continuous training can induce similar improvements in choice response time (25). It can be deduced that improvements in HIIT response time are not achieved overnight, but long-term exercise can lead to alterations in response time. This is primarily because our exercise program also included some resistance training content. Resistance training exercises the neuromuscular system and increases the brain’s ability to mediate the rapid response of the muscles, thereby increasing the performance of the choice response.

### Improvement in muscle strength

Muscle strength is highly vulnerable in patients with substance use disorders (19, 29, 38), and the inactive lifestyle of drug abstinents may indirectly lead to decreased muscle strength. In this study, push-ups and grip strength were used to evaluate the muscle strength of substance use disorder. After 8 months of HIIT, it was found that the push-up performance of the HIIT group increased from baseline (46.75 ± 11.29) to 8 months (59.36 ± 11.94). There was a steady increase, but in contrast, the increase in grip strength scores from baseline (39.66 ± 6.28) to 8 months (41.82 ± 6.64) was not as pronounced. This may be attributed to the fact that during exercise intervention, large muscle groups exercise more than those of the forearm or fingers, and as a result, push-up performance significantly increases.

### Balance and responsiveness improvements

Neuroimaging studies have demonstrated that methamphetamine can damage most cerebral regions in the central nervous system of the human brain, including the ventral tegmental area of the midbrain, nucleus accumbens, prefrontal cortex, locus coeruleus, hippocampus, striatum. Etc., resulting in pathological changes similar to Alzheimer’s disease and Parkinson’s disease, affecting the movement, learning ability, and memory of abusers, eventually leading to movement disorders, including balance disorders, decreased motor coordination, decreased sensitivity and flexibility, etc. (39–42). Loss of balance and functions owing to brain injury and possible muscle wasting may also occur. In our study, we not only confirmed that 8 months of HIIT can increase muscle strength, but it also significantly improved the scores in the test of standing on one foot with eyes closed (15.46 ± 11.76——33.61 ± 24.64). During the withdrawal period, given that the individual is primarily engaged in routine rehabilitation care, he/she is less exposed to high-intensity anaerobic and aerobic training, such as emergency stop and sudden start, rapid change of direction, and adjustment of body orientation in basketball training, which can improve performance. Furthermore, the individual’s ability for body balance and sensitivity (22.54 ± 2.74——27.93 ± 3.42) was increased.

### Weight, body fat percentage, waist circumference, and flexibility

Generally speaking, MA abuse can easily lead to weight loss, predominantly due to loss of appetite during the abuse process (43–45). Studies have shown that MA addicts have lower body fat percentages than healthy controls (46). On the contrary, during the withdrawal period, their body fat percentage significantly increases in view of appetite reduction and metabolism elimination induced by drug abuse (34, 47, 48). If no measures are taken to suppress body weight, fat percentages, etc., it may make recovery more challenging and lead to more severe chronic conditions. Herein, after HIIT training, body weight, body fat percentage, and waist circumference were significantly decreased, and the related flexibility was also improved, leading to a healthier body.

### Changes in quality of life and cravings

Consequences of drug use include a severe deterioration in the quality of life and physical and mental health. Therefore, drug addiction rehabilitation should not only focus on pure detoxification but should also strive to restore the patient’s quality of life and physical and mental health (49). The use of aerobics combined with resistance exercise or yoga has been found to improve the quality of life of substance use disorder (50–52); however, some studies have reported that the effect of exercise was not immediately apparent (53). Therefore, the type of exercise, intensity, and so on may be the determining factors in whether exercise results in positive outcomes. This study was based on the premise that HIIT is the ideal exercise method to improve the quality of life of people with chronic diseases (27, 28, 30) and attempted to explore this approach from a newer and more comprehensive perspective. The effect of exercise on sleep quality is obvious. Through research, we found that the total score of PSQI decreased, which to some extent explains the improvement of exercise on sleep, which will lay a foundation for the improvement of the overall health level of patients with substance use disorders.

This study used long-term, high-intensity exercise to explore its impact on improving the quality of life of substance use disorder. To the best of our knowledge, this is the first study to explore the effects of long-term HIIT based on health-related quality and comprehensive physical fitness parameters. The results are promising; not only did physical fitness indicators improve, but so did the quality of life and craving levels. This may be because HIIT can comprehensively improve the physical fitness level of substance use disorder, giving individuals a sense of accomplishment and active control over their bodies, thereby improving self-efficacy, elevating their mental state, and increasing their responsiveness to drugs. This may be due to the fact that HIIT has improved the physical health of patients with substance use disorders and the confidence of individuals to abstain from drug use, thus improving the quality of life of patients with drug use disorders. Among them, non-confrontational basketball practice has the characteristics of high intensity but not intense, which is more suitable for the existing lower sports quality and physical function level of patients with substance use disorders, and can also increase the subject’s team cooperation ability and social interaction ability to a certain extent, which has a good effect in improving the quality of life.

### Correlation between physiological indexes and craving degree and quality of life

One of the innovations of this study is to construct the correlation between the physiological and biochemical indicators of patients with substance use disorders and the degree of craving and quality of life for the first time. Through analysis, we found that VO2max, which represents the individual’s heart and lung health level, has a high correlation with cue-induced craving and quality of life related to physiological aspects. At the same time, HIIT can improve the status of all indicators, This is similar to Gry Bang-Kittilsen et al. who found that HIIT can improve the depression level of schizophrenic patients by regulating their VO2max (54). This may be because exercise can improve the level of cardiovascular system and then enhance the self-efficacy of individuals, increase their confidence to quit drug addiction, and then improve the degree of craving and increase the quality of life. At the same time, there is a correlation between PCS and MCS, which also proves that the internal consistency of the scale is good and the description of quality of life is accurate. At the same time, we found that standing on one foot with eyes closed was correlated with psychologically related quality of life, which may be due to the damage of brain central nervous system related to balance ability caused by drug use, and then led to the impairment of psychological function. However, the improvement effect of sports is common and obvious.

### Limitations and deficiencies

Notably, this study also has some limitations which need to be taken into account. To begin with, we could not observe the continuation of the effect of long-term exercise intervention without follow-ups. Our research group intends to increase the frequency of follow-ups in future studies to observe the effect more comprehensively. Secondly, the female population was not included. This is mostly due to the fact that the majority of person who uses drugs in China are male, and sampling was relatively simple in the past. Our team will aim to expand the sample population to include females in future studies. Lastly, this study only explored improvements in quality of life resulting from physical fitness. In the future, we aim to expand the discussion to neuroendocrine and immune mechanisms and examine them from various aspects and perspectives.

## Conclusion

Eight months of HIIT can comprehensively improve the physical fitness level of male methamphetamine abstinent patients, improve health-related quality of life, reduce drug cravings, and lay the foundation for a better return to society.

## Data availability statement

The original contributions presented in the study are included in the article/Supplementary material, further inquiries can be directed to the corresponding author.

## Ethics statement

This human study was reviewed and approved by the Ethical Review Committee of Hunan Normal University (batch number: 2020–233). The participants provided written informed consent to participate.

## Author contributions

JT wrote the manuscript and participated in the exercise intervention and data analysis. JW, YG, WT, and CL participated in exercise intervention and data collection. LZ designed and directed the study. All authors contributed to the article and approved the submitted version.

## Conflict of interest

The authors declare that the research was conducted in the absence of any commercial or financial relationships that could be construed as a potential conflict of interest.

## Publisher’s note

All claims expressed in this article are solely those of the authors and do not necessarily represent those of their affiliated organizations, or those of the publisher, the editors and the reviewers. Any product that may be evaluated in this article, or claim that may be made by its manufacturer, is not guaranteed or endorsed by the publisher.
