# Supplementary material for: Effects of 8 months of high-intensity interval training on physical fitness and health-related quality of life in substance use disorder
Source: Front Psychiatry. 2023 Aug 9;14:1093106. doi: 10.3389/fpsyt.2023.1093106 (PMC10445760; doi:10.3389/fpsyt.2023.1093106)
Supplement: Supplementary file 1 [file Table_1.docx]

Table S1 Correlation analysis of cue-induced craving and health-related quality of life of various physiological indicators

|  | Heart rate | Systolic blood pressure | Systolic blood pressure | Weight | Waistline | BMI | VO_2_max | Grip | Reaction time | Reaction time | Standing with eyes closed | Sitting forward bend | Quadrant jump | Total cholesterol | Triglyceride | Blood sugar | PSQI | VAS | PCS | MCS |
| --- | --- | --- | --- | --- | --- | --- | --- | --- | --- | --- | --- | --- | --- | --- | --- | --- | --- | --- | --- | --- |
| Heart rate | *r=*1 | *r=-*0.026  *p*=0.849 | *r=*0.107  *p*=*0.433* | *r=0.054*  **  *p<0.*001 | *r=*-0.268*  *p<0.*05 | *r=*0.017  *p=*0.904 | *r=*0.139  *p*=0.036 | *r=*-0.026  *p*=0.850 | *r=-*0.081  *p*=0.551 | *r=*0.121  *p*=0.374 | *r=-*0.118  *p*=0.387 | *r=-*0.009  *p*=0.946 | *r=*0.097  *p*=0.477 | *r=*0.117  *p*=0.547 | *r=*0.219  *p*=0.614 | *r=*0.124  *p*=0.078 | *r=*0.179  *p*=0.870 | *r=*0.048  *p*=0.728 | *r=*0.143  *p*=0.293 | *r=*0.164  *p*=0.227 |
| Systolic blood pressure |  | *r=*1 | *r=*0.407**  *p<0.*01 | *r=*0.174  *p*=0.200 | *r=*-0.054  *p*=0.692 | *r=*0.255  *p*=0.057 | *r=-*0.0.015  *p*=0.910 | *r=*0.199  *p*=0.141 | *r=*0.138  *p*=0.311 | *r=*0.079  *p*=0.562 | *r=-*0.140  *p*=0.304 | *r=*0.235  *p*=0.081 | *r=-*0.274*  *p<0.*05 | *r=*0.248  *p*=0.054 | *r=*0.874  *p*=0.079 | *r=*0.259  *p*=0.147 | *r=*0.679  *p*=0.358 | *r=-*0.475  *p*=0.729 | *r=-*0.202  *p*=0.135 | *r=-*0.131  *p*=0.335 |
| Systolic blood pressure |  |  | *r=*1 | *r=*0.011  *p*=0.935 | *r=*-0.091  *p*=0.504 | *r=*0.117  *p*=0.392 | *r=*0.037  *p*=0.787 | *r=-*0.015  *p*=0.915 | *r=-*0.042  *p*=0.758 | *r=*0.143  *p*=0.294 | *r=-*0.033  *p*=0.807 | *r=*0.113  *p*=0.406 | *r=-*0.261  *p*=0.052 | *r=*0.584  *p*=0.256 | *r=*0.361  *p*=0.195 | *r=*0.147  *p*=0.061 | *r=*0.247  *p*=0.145 | *r=*0.039  *p*=0.773 | *r=-*0.114  *p*=0.403 | *r=-*0.23104  *p*=0.406 |
| Weight |  |  |  | *r=*1 | *r=*-0.200  *p*=0.140 | *r=*0.053  *p*=-0.700 | *r=*0.109  *p*=0.402 | *r=-*0.106  *p*=0.906 | *r=-*0.066  *p*=0.628 | *r=*0.150  *p*=0.270 | *r=-*0.054  *p*=0.691 | *r=-*0.002  *p*=0.991 | *r=-*0.084  *p*=0.539 | *r=*0.248  *p*=0.075 | *r=*0.951  *p*=0.841 | *r=*0.369  *p*=0.930 | *r=*0.842  *p*=0.417 | *r=-*0.036  *p*=0.790 | *r=-*0.085  *p*=0.534 | *r=-*0.117  *p*=0.392 |
| Waistline |  |  |  |  | *r=*1 | *r=-*0.034  *p=*0.803 | *r=-*0.221  *p*=0.101 | *r=*0.004  *p*=0.977 | *r=*0.031  *p*=0.818 | *r=*0.041  *p*=0.763 | *r=*0.027  *p*=0.846 | *r=-*0.136  *p*=317 | *r=-*0.017  *p*=0.899 | *r=*0.462  *p*=0.641 | *r=*0.148  *p*=0.752 | *r=*0.1627  *p*=0.320 | *r=*0.520  *p*=0.104 | *r=*0.251  *p*=0.062 | *r=-*0.001  *p*=0.993 | *r=*0.157  *p*=0.246 |
| BMI |  |  |  |  |  | *r=*1 | *r=*0.164  *p*=0.226 | *r=*0.155  *p*=0.252 | *r=-*0.012  *p*=0.929 | *r=*0.229  *p*=0.909 | *r=*0.128  *p*=0.348 | *r=*0.10  *p*=0.428 | *r=-*0.126  *p*=0.353 | *r=*0.63  *p*=0.742 | *r=*0.478  *p*=0.442 | *r=*0.713  *p*=0.358 | *r=*0.148  *p*=0.247 | *r=-*0.187  *p*=0.167 | *r=*0.104  *p*=0.444 | *r=-*0.033  *p*=0.807 |
| VO_2_max |  |  |  |  |  |  | *r=*1 | *r=*0.245  *p*=0.068 | *r=*0.110  *p*=0.419 | *r=-*0.066  *p*=0.627 | *r=-*0.044  *p*=0.749 | *r=*0.040  *p*=0.768 | *r=*0.001  *p*=0.997 | *r=*0.143  *p*=0.214 | *r=*0.027  *p*=0.847 | *r=*0.045  *p*=0.713 | *r=*0.071  *p*=0.701 | *r=-*0.434  **  *p<0.*001 | *r=*0.425  **  *p<0.*001 | *r=*0.118  *p*=0.385 |
| Grip |  |  |  |  |  |  |  | *r=*1 | *r=*0.019  *p*=0.888 | *r=-*0.231  *p*=0.087 | *r=-*0.0142  *p*=0.298 | *r=*0.057  *p*=0.679 | *r=*0.092  *p*=0.458 | *r=*0.141  *p*=0.543 | *r=*0.169  *p*=0.767 | *r=*0.748  *p*=0.363 | *r=*0.078  *p*=0.874 | *r=-*0.071  *p*=0.605 | *r=*0.056  *p*=0.683 | *r=-*0.097  *p*=0.478 |
| Push ups |  |  |  |  |  |  |  |  | *r=*1 | *r=*0.037  *p*=0.788 | *r=*0.119  *p*=0.380 | *r=*0.041  *p*=0.763 | *r=-*0.240  *p*=0.704 | *r=*0.241  *p*=0.143 | *r=*0.041  *p*=0.367 | *r=*0.479  *p*=0.741 | *r=*0.014  *p*=0.796 | *r=-*0.031  *p*=0.7820 | *r=-*0.262  *p*=0.051 | *r=-*0.010  *p*=0.941 |
| Reaction time |  |  |  |  |  |  |  |  |  | *r=*1 | *r=*0.038  *p*=0.781 | *r=*0.033  *p*=0.810 | *r=-*0.020  *p*=0.763 | *r=*0.041  *p*=0.138 | *r=*0.147  *p*=0.684 | *r=*0.295  *p*=0.081 | *r=*0.149  *p*=0.284 | *r=*0.033  *p*=0.809 | *r=*0.108  *p*=0.430 | *r=-*0.010  *p*=0.941 |
| Standing with eyes closed |  |  |  |  |  |  |  |  |  |  | *r=*1 | *r=*0.038  *p*=0.781 | *r=-*0.201  *p*=0.877 | *r=*0.240  *p*=0.081 | *r=*0.874  *p*=0.247 | *r=*0.123  *p*=0.658 | *r=*0.279  *p*=0.141 | *r=*0.033  *p*=0.809 | *r=*0.108  *p*=0.430 | *r=*0.283*  *p<0.*05 |
| Sitting forward bend |  |  |  |  |  |  |  |  |  |  |  | *r=*1 | *r=*0.28  *p*=0.837 | *r=*0.128  *p*=0.441 | *r=*0.781  *p*=0.984 | *r=*0.369  *p*=0.401 | *r=*0.753  *p*=0.420 | *r=*0.073  *p*=0.590 | *r=-*0.143  *p*=0.293 | *r=*0.025  *p*=0.857 |
| Quadrant jump |  |  |  |  |  |  |  |  |  |  |  |  | *r=*1 | *r=*0.713  *p*=0.810 | *r=-*0.029  *p*=0.613 | *r=*0.041  *p*=0.138 | *r=*0.179  *p*=0.634 | *r=*0.074  *p*=0.589 | *r=*0.009  *p*=0.112 | *r=*0.101  *p*=0.458 |
| Total cholesterol |  |  |  |  |  |  |  |  |  |  |  |  |  | *r=*1 | *r=*0.53  *p*=0.819 | *r=-*0.420  *p*=0.443 | *r=*0.147  *p*=0.179 | *r=*0.179  *p*=0744 | *r=*0.168  *p*=0.658 | *r=*0.748  *p*=0.734 |
| Triglyceride |  |  |  |  |  |  |  |  |  |  |  |  |  |  | *r=*1 | *r=*0.087  *p*=0.410 | *r=-*0.080  *p*=0.873 | *r=*0.279  *p*=0.647 | *r=*0.141  *p*=0.670 | *r=*0.179  *p*=0.914 |
| Blood sugar |  |  |  |  |  |  |  |  |  |  |  |  |  |  |  | *r=*1 | *r=-*0.063  *p*=0.767 | *r=*0.146  *p*=0.634 | *r=*0.879  *p*=0.673 | *r=*0.134  *p*=0.584 |
| PSQI |  |  |  |  |  |  |  |  |  |  |  |  |  |  |  |  | *r=*1 | *r=*0.741  *p*=0.624 | *r=*0.056  *p*=0.587 | *r=*0.179  *p*=0.638 |
| **VAS** |  |  |  |  |  |  |  |  |  |  |  |  |  |  |  |  |  | *r=*1 | *r=*0.041  *p*=0.764 | *r=*0.095  *p*=0.486 |
| **PCS** |  |  |  |  |  |  |  |  |  |  |  |  |  |  |  |  |  |  | *r=*1 | *r=*0.459  **  *p<0.*001 |
| **MCS** |  |  |  |  |  |  |  |  |  |  |  |  |  |  |  |  |  |  |  | *r=*1 |
